# Macroscopic hematuria in children with autosomal dominant polycystic kidney disease — report from four European tertiary centers

**DOI:** 10.1007/s00431-026-07022-1

**Published:** 2026-05-07

**Authors:** Tomáš Seeman, Květa Bláhová, Filip Fencl, Ulrike John-Kroegel, Friederike Weigel, Terezie Šuláková, Ludmila Podracká, Janusz Feber

**Affiliations:** 1 Department of Pediatrics, 2nd Medical Faculty Charles University Prague, Prague, Czech Republic; 2 Department of Pediatrics, Medical Faculty of University Ostrava, University Hospital Ostrava, Ostrava, Czech Republic; 3 Division of Pediatric Nephrology, University Children´s Hospital Jena, Jena, Germany; 4 Department of Pediatrics, Medical Faculty, Comenius University and National Institute of Children’s Diseases, Bratislava, Slovakia; 5 Children’s Hospital of Eastern Ontario, University of Ottawa, Ottawa, ON Canada

**Keywords:** Autosomal dominant polycystic kidney disease, Children, Macroscopic hematuria, Hypertension

## Abstract

In adults with autosomal dominant polycystic kidney disease (ADPKD), episodes of macroscopic hematuria are relatively common and are predictors of hypertension. We hypothesized that a history of macroscopic hematuria will be associated with hypertension also in children with ADPKD. We retrospectively analyzed 289 children (median age 10.2 years) with ADPKD who were followed in four European tertiary nephrology centers. The presence of ≥ 1 episode of macroscopic hematuria (MaHu) defines a group of MaHu+ patients; the absence defines a group of MaHu− patients. Hypertension was defined as the use of antihypertensive drugs upon the last investigation. Macroscopic hematuria occurred in 7.2% of children (*n* = 21). It was the second most frequent presenting symptom of ADPKD (11 out of 51 children). The etiology of macroscopic hematuria included ruptured cyst, urinary tract infection or urolithiasis. The prevalence of hypertension at the last follow-up in MaHu+ children was significantly higher than in MaHu− children (14.3% vs. 3.1%, *p* = 0.017). The proportion of patients with elevated blood pressure ≥ 75th percentile was significantly higher in patients with hematuria as compared with patients with no hematuria (95% vs. 64%, *p* = 0.002). Children from the MaHu+ group had a significantly higher number of cysts when compared to the MaHu− group (20 vs. 10 cysts, *p* = 0.038). There were no significant differences between MaHu+ vs. MaHu− groups in other variables.

*Conclusion*: In children with ADPKD, a history of macroscopic hematuria is associated with an increased risk of developing hypertension and/or elevated blood pressure.

**Table:** 

**What is Known:** • *Adults with autosomal dominant polycystic kidney disease have often episodes of macroscopic hematuria that predict development of hypertension.*	
**What is New:** • *In children with autosomal dominant polycystic kidney disease, macroscopic hematuria is rare but is also associated with an increased risk of developing hypertension.*

**What is Known:**

• *Adults with autosomal dominant polycystic kidney disease have often episodes of macroscopic hematuria that predict development of hypertension.*

**What is New:**

• *In children with autosomal dominant polycystic kidney disease, macroscopic hematuria is rare but is also associated with an increased risk of developing hypertension.*

## Introduction

Autosomal dominant polycystic kidney disease (ADPKD) is the most common hereditary kidney disease that affects 1 in 500–1000 individuals [1–3]. In adults, episodes of macroscopic hematuria are relatively common (10–37%), and a recent study from the Spanish national ADPKD registry showed that it is also an independent predictor of arterial hypertension [4]. On the contrary, in children, there is little information on the prevalence, factors, and long-term associations of macroscopic hematuria [5, 6]. We hypothesized that a history of macroscopic hematuria will be associated with hypertension also in children with ADPKD. We aimed to study the prevalence and risk factors associated with macroscopic hematuria in a large cohort of children with ADPKD.

## Materials and methods

The records from all 377 pediatric patients diagnosed with ADPKD from 4 Central European tertiary pediatric nephrology centers were retrospectively reviewed for the patients’ eligibility for the study. The inclusion criteria for eligibility were (1) diagnosis of ADPKD (based on positive family history of ADPKD in a parent, combined with the presence of at least two kidney cysts) [7] and (2) information on the presence or absence of an episode of macroscopic hematuria in the past medical history and (3) complete laboratory and clinical investigations (kidney ultrasound, office blood pressure, urinalysis, and serum creatinine).

In total, 289 children (median age 10.2 years, IQR 5.8–14.9) fulfilled the inclusion criteria and were retrospectively investigated. The presence of ≥ 1 episode of macroscopic hematuria (clinically confirmed) defined a group of MaHu+ patients; the absence of macroscopic hematuria defined a group of MaHu− patients.

### Hypertension

Hypertension was defined as the use of antihypertensive drugs during our last investigation, similarly to adults [4]. In addition, we defined elevated blood pressure (BP) as systolic or diastolic BP ≥ 75th percentile (≥ 0.674 *Z*-scores) for age and height based on office BP normative data [8], as recommended by the most recent KDIGO ADPKD guidelines [2].

#### Office blood pressure

Office BP was measured using a mercury sphygmomanometer or an oscillometric manometer through standard techniques [6]. Office systolic and diastolic BP index was calculated using the patient’s systolic and diastolic BP divided by the 95th percentile [9].

#### Ambulatory blood pressure

ABPM studies were carried out in a subgroup of children using oscillometric SpaceLabs 90207 or 90217 monitors (SpaceLabs Medical, Redmond, WA), using proper techniques [10]. The ambulatory blood pressure index was calculated as the mean daytime and nighttime systolic and diastolic BP, divided by the 95th percentile.

### Renal function and proteinuria

Estimated glomerular filtration rate (eGFR) was estimated using the Schwartz formula [11], serum creatinine levels (Jaffe method), or using the new Schwartz formula [12] from the serum creatinine levels, measured by the enzymatic method (Advia 1800, Siemens, USA). Chronic kidney disease (CKD) stages were classified according to the KDIGO 2024 Guidelines [13].

Urine collected over 24 h was tested for quantitative measurements of total protein (Biuret method) in the majority of children (*n* = 160), whereas proteinuria was assessed during the first morning urine (*n* = 90) or using reagent strip urinalysis for total protein (*n* = 49). Proteinuria was defined as urinary protein excretion greater than 96 mg/m^2^/24 h in urine, or greater than 20 mg/mmol creatinine, or total protein ≥ 1 on reagent strip urinalysis [14].

### Kidney ultrasound

Kidney ultrasound was performed using Toshiba Aplio XG (Tochigi, Japan) or Toshiba Power Vision 6000 (Otawara, Japan), to figure out the length of each kidney and the number of cysts in each kidney. Kidney length was compared with values expected for body height [15]. Severe cystic involvement was defined as ≥ 10 cysts in both kidneys [16].

### Data analysis

Data was analyzed within Python with the following packages: Table one (group comparison), Dabest (estimation statistics), and Pingouin (statistics). All continuous variables are shown as medians and interquartile ranges, with some variables showing abnormal distribution on the test for normality of distribution (Shapiro Wilk test). Comparison between groups (macroscopic hematuria vs no−hematuria) was performed using Chi−Square statistics (categorical variables) and the Wilcoxon non−parametric test. Additionally, estimation statistics with 95% confidence interval of the difference between groups and permutation *p*−value were used for selected parameters which were identified as statistically significantly different on group comparisons (prevalence of hematuria episodes, number of cysts, office blood pressure indices, ABPM indices).

## Results

### Macroscopic hematuria

At least one episode of macroscopic hematuria (clinically confirmed) occurred in 21/289 (7.2%) of children. Macroscopic hematuria was the first symptom of ADPKD leading to diagnosis in 11 children (3.8%) and was the second most frequent presenting symptom of ADPKD (11 out of 51 children) following abdominal pain. Macroscopic hematuria occurred in 11 normotensive children, one child was already treated by antihypertensive drug at the time of hematuria attack and the timing of BP elevation in relation to the hematuria episode was unknown in 9 children.

The age recorded at the first episode of macroscopic hematuria was between 4 and 17 years in 16 children and unknown in 5 children. The median time interval between the first episode of macroscopic hematuria and investigations was 6.1 years (range 0.5–12), 4.4 years (range 0.3–9.5) in the MaHu− group (NS in comparison to MaHu+ group) and 4.6 years (0.3–12) in the entire cohort. The etiology of macroscopic hematuria was a ruptured cyst, urinary tract infection (five each), urolithiasis (two), and unknown in 9 children.

### Demographic, clinical, and laboratory data

The demographic, clinical, laboratory, and molecular genetic data are summarized in Table 1.

**Table 1:** Demographic, clinical, laboratory, and molecular genetic data of children with ADPKD with and without a history of macroscopic hematuria

Parameter	All	No macroscopic hematuria	Macroscopic hematuria	*p*-value
Nr	289	268	21	
Females, *n* (%)	138 (47.92)	131 (49.06)	7 (33.33)	0.245
Age at exam (years)	10.23 [5.79, 14.86]	10.11 [5.56, 14.27]	15.80 [9.68, 17.55]	**0.001**
Weight (kg)	35.20 [20.80, 56.00]	34.00 [19.85, 55.00]	60.00 [45.00, 71.00]	**< 0.001**
Height (cm)	143.00 [115.75, 168.00]	141.00 [114.50, 166.00]	170.00 [140.00, 182.00]	**0.002**
BMI (kg/m2)	17.35 [15.40, 20.17]	17.19 [15.37, 19.84]	20.53 [17.53, 21.04]	**0.019**
BMI *Z*-score (SDS)	− 0.20 [− 0.80, 0.70]	− 0.20 [− 0.80, 0.70]	− 0.20 [− 0.50,0.30]	0.976
S-crea (μmol/l)	53.00 [41.00, 68.00]	52.00 [40.75, 67.00]	69.50 [58.75, 82.75]	**< 0.001**
GFR (ml/min/1.73 m^2^)	118.05 [104.32, 136.77]	118.80 [104.07, 137.75]	112.50 [107.04, 117.95]	0.123
RK length (mm)	96.00 [82.75, 110.00]	96.00 [82.00, 110.00]	113.00 [85.50, 123.00]	**0.018**
RK length (SDS)	1.03 [0.24, 1.77]	1.04 [0.24, 1.75]	0.97 [0.32, 2.36]	0.577
LK length (mm)	98.00 [85.00, 112.00]	97.00 [84.75, 110.00]	113.00 [93.00, 124.50]	**0.012**
LK length (SDS)	1.14 [0.32, 2.07]	1.11 [0.32, 2.07]	1.43 [0.48, 2.20]	0.456
Nr of cysts (*n*)	10.00 [7.00, 20.00]	10.00 [6.00, 20.00]	20.00 [7.00, 30.00]	**0.038**
Nr of cysts > 10, *n* (%)	145 (51.06)	133 (50.57)	12 (57.14)	0.724
U-conc decreased, *n* (%)	33 (55.00)	30 (54.55)	3 (60.00)	1
Proteinuria, *n* (%)	74 (36.10)	70 (36.65)	4 (28.57)	0.75
PKD1 P/LP variants (%)	68	68	67	0.89
PKD2 P/LP variants (%)	10	10	5	0.78

*BMI* body mass index, *S-crea* serum creatinine, *GFR* glomerular filtration rate, *RK* right kidney, *LK* left kidney, *SDS* standard deviation score, *U-con.decreased* urinary concentrating capacity decreased, *P/LP* pathogenic/likely pathogenic

All continuous variables are presented as median and interquartile range

### Hypertension

Eleven patients (3.8%) were treated by antihypertensive drugs (angiotensin-converting enzyme inhibitors (ACEIs) in 10 patients). Ramipril was the most used ACEI (*n* = 8), with two patients being treated with combination antihypertensive therapy (diuretic, beta-blocker).

**Fig. 1:**
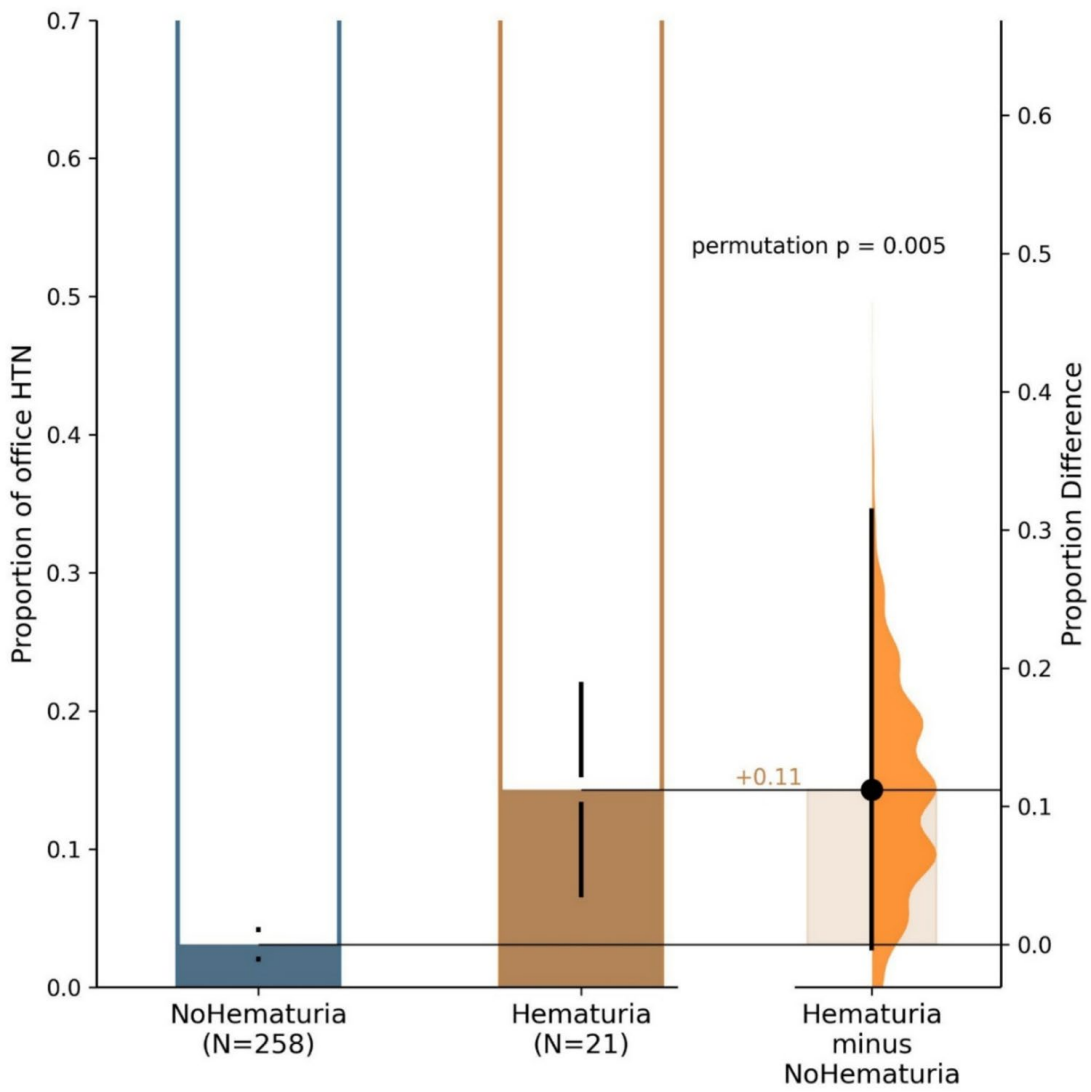
Prevalence of hypertension defined by the use of antihypertensive medications at the last follow−up in children with and without a history of macroscopic hematuria. The two bar graphs represent proportions of patients with hypertension in hematuria and non−hematuria groups, the third bar with violin plot shows the proportion difference with 95% confidence interval of the difference in proportions between groups (as evaluated by the estimation statistics)

**Fig. 2:**
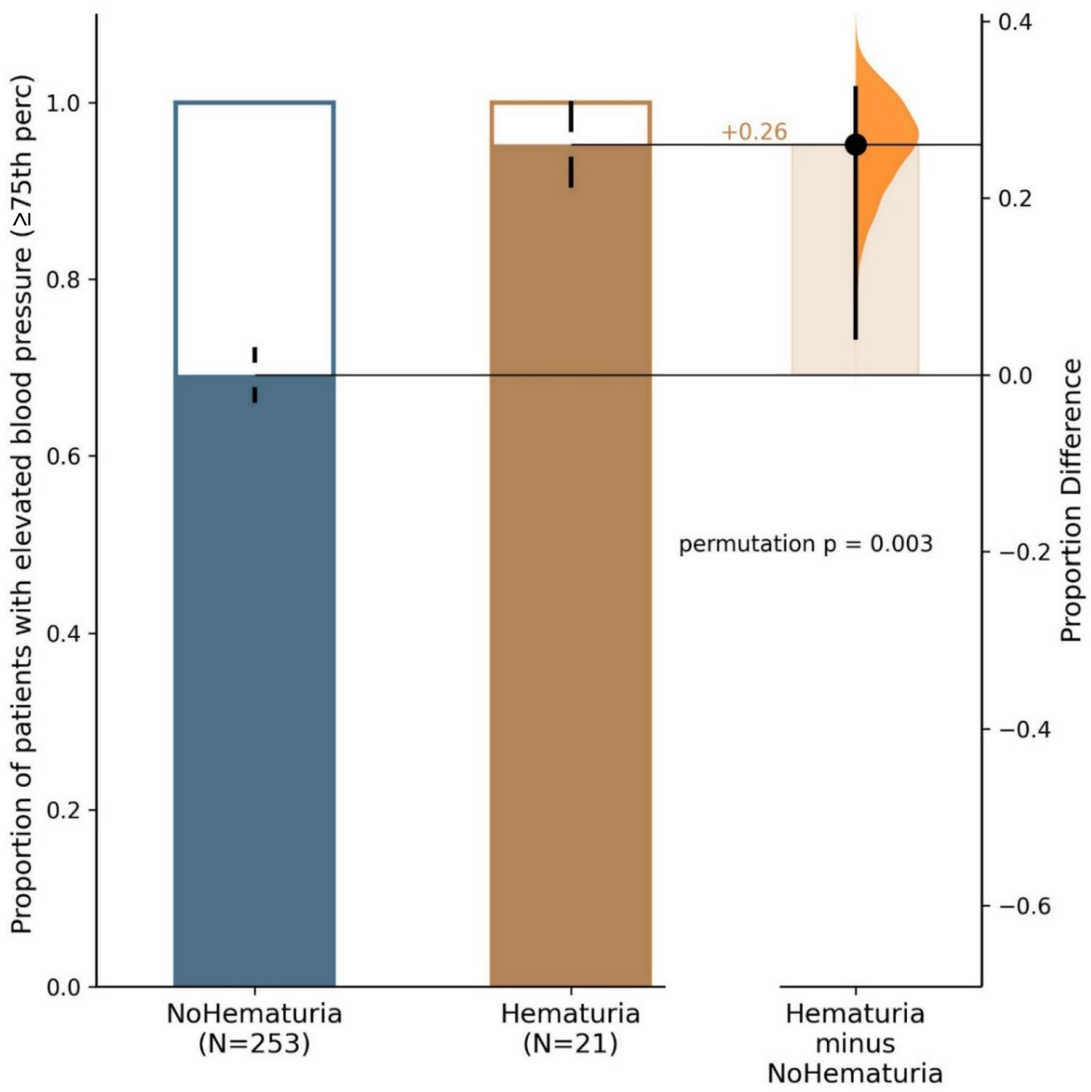
Prevalence of elevated blood pressure defined as the blood pressure *z*−score ≥75^th^ percentile (≥0.674 *Z*−score) at the last follow−up in children with and without a history of macroscopic hematuria. The two bar graphs represent proportions of patients with hypertension in hematuria and non−hematuria groups, the third bar with violin plot shows the proportion difference with 95% confidence interval of the difference in proportions between groups (as evaluated by the estimation statistics)

The prevalence of hypertension defined by the use of antihypertensive medications during the last follow-up visit in children with a history of macroscopic hematuria was significantly higher than in children without a history of macroscopic hematuria (14.3% vs. 3.1%, *p* = 0.017, Table 2, Fig. 1). Similarly, the proportion of patients with elevated blood pressure (BP equal to or above the 75th percentile) was significantly higher in patients with hematuria as compared with patients with no hematuria (95% vs. 64%, *p* = 0.002, Fig. 2).

**Table 2:** Office and ambulatory blood pressure of children with ADPKD with and without a history of macroscopic hematuria

Parameter	All	No macroscopic hematuria	Macroscopic hematuria	*p*-value
Nr	289	268	21	
SBP (mmHg)	110.00 [100.00, 120.00]	110.00 [100.00, 120.00]	122.00 [115.00, 132.00]	**< 0.001**
DBP (mmHg)	65.00 [60.00, 75.00]	65.00 [58.00, 73.00]	80.00 [64.00,82.00]	**0.001**
SBP index	0.92 [0.84, 0.97]	0.92 [0.84, 0.97]	0.94 [0.87, 1.02]	0.064
DBP index	0.84 [0.75, 0.93]	0.84 [0.75, 0.93]	0.89 [0.78, 1.01]	**0.048**
Hypertension, *n* (%)	11 (3.94)	8 (3.10)	3 (14.29)	**0.041**
Day SBP (mmHg)	117.50 [110.00, 124.00]	117.00 [109.00, 124.00]	126.00 [121.00, 128.50]	**0.005**
Day SBP index	0.90 [0.86, 0.95]	0.90 [0.86, 0.95]	0.94 [0.93, 0.97]	**0.038**
Day DBP (mmHg)	70.50 [66.00, 76.00]	71.00 [66.00, 76.00]	69.00 [66.50, 72.50]	0.767
Day DBP index	0.84 [0.80, 0.91]	0.85 [0.80, 0.91]	0.84 [0.81, 0.87]	0.847
Night SBP (mmHg)	105.00 [98.00, 112.00]	103.50 [98.00, 112.00]	113.00 [109.00, 121.00]	**0.002**
Night SBP index	0.92 [0.87, 0.96]	0.92 [0.87, 0.96]	0.94 [0.90, 1.00]	0.07
Night DBP (mmHg)	59.00 [54.00, 63.00]	58.00 [54.00, 62.00]	62.00 [59.50, 66.50]	0.037
Night DBP index	0.89 [0.82, 0.95]	0.88 [0.82, 0.95]	0.94 [0.90, 1.00]	0.05

*SBP* systolic blood pressure, *DBP* diastolic blood pressure

The relationship between the presence/absence of a history of macroscopic hematuria and other variables is described in Table 1.

### Blood pressure

#### Office BP

Diastolic office BP index during the last visit was significantly higher in the MaHu+ group, in comparison to the MaHu− group (Table 2). Systolic office BP index during the last visit was not significantly higher in the MaHu+ group, in comparison to the MaHu− group (Table 2).

#### Ambulatory BP

A subgroup of 157 children had their ABPM performed during their last follow-up. The systolic daytime ambulatory BP index during their last visit was significantly higher in the MaHu+ group, in comparison to the MaHu− group (Table 2). Systolic nighttime and diastolic daytime and nighttime ambulatory BP indices during the last visit were not significantly higher in the MaHu+ group when compared to the MaHu− group (Table 2).

### Kidney cystic involvement and function

**Fig. 3:**
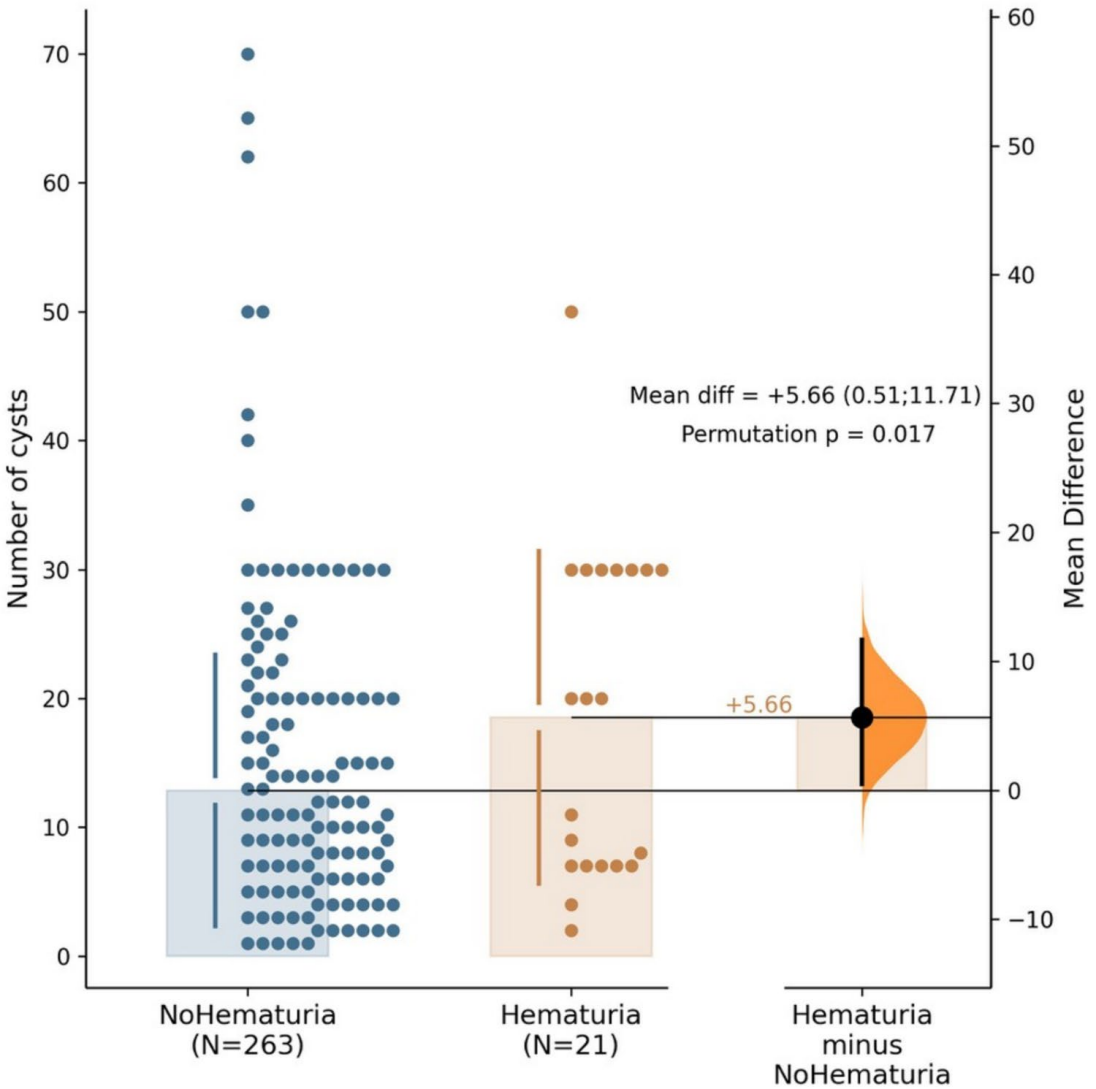
Total number of kidney cysts in children with and without a history of macroscopic hematuria. The bar with violin plot shows the difference between the number of cysts in both groups with 95% confidence interval (as evaluated by the estimation statistics)

Children from the MaHu+ group had a significantly higher number of cysts in both kidneys than the children from the MaHu− group (20 vs. 10 cysts, *p* = 0.038, Table 1, Fig. 3).

There were no significant differences between MaHu+ vs. MaHu− groups in kidney length, percentage of severe cystic involvement, eGFR, or pathological proteinuria (Table 1).

#### Microscopic hematuria

Only two (0.7%) children had microscopic hematuria during their last investigation (one child in each group).

#### DNA analysis

In 60 children, a DNA analysis was performed with a positive result. It showed 55 children with a likely pathogenic or pathogenic variant in the *PKD1* gene, along with 5 children having the *PKD2* gene. There was no difference in the prevalence of macroscopic hematuria between children with PKD1 and PKD2 variants. The occurrence of PKD1 variants did not differ significant between the groups with and without a history of macroscopic hematuria.

## Discussion

In our multicenter international retrospective study, we could demonstrate for the first time that also in children with ADPKD, a history of macroscopic hematuria is associated with hypertension and/or elevated blood pressure later in life. Moreover, macroscopic hematuria was associated with a higher number of kidney cysts—a marker of more severe cystic kidney disease.

Adults with ADPKD suffer relatively common (10–37%) episodes of macroscopic hematuria [16], and a recent study from the Spanish national ADPKD registry showed that it is also an independent predictor of arterial hypertension [2]. On the contrary, in children, there is little information on the prevalence, factors, and long-term associations of episodes of macroscopic hematuria [5, 6]. The prevalence of macroscopic hematuria in pediatric studies ranges from 3 to 15% [5, 6, 17, 18], which is considerably lower when compared to adults. The 7% prevalence of macroscopic hematuria in our study is in line with earlier pediatric studies.

The most common etiologies of macroscopic hematuria in ADPKD patients are hemorrhagic rupture of cysts, infections in the cysts and nephrolithiasis [2, 16]. The risk factors associated with macroscopic hematuria in pediatric studies are enlarged kidneys and adolescent age when compared to the younger pediatric population [6, 18]. Surprisingly, children with very-early onset (VEO) ADPKD had a similar prevalence of macroscopic hematuria compared to children with later onset ADPKD in one study [18].

### Hypertension

Hypertension is one of the early complications of ADPKD, being detected in 50–80% of adult patients and in 27–37% of pediatric patients [1–3, 7, 8, 19, 20]. More severe cystic kidney involvement, PKD1 gene variants, eGFR, and male sexual orientation are the main risk factors for developing hypertension [16, 19, 21]. In a Spanish registry study, the authors show that a history of macroscopic hematuria is another risk factor for arterial hypertension in adults [4]. Furthermore, in a North American study, macroscopic hematuria was independently associated with faster progression of chronic kidney disease [16]. We found an absence of similar pediatric ADPKD studies relating to the association of macroscopic hematuria and later onset hypertension. Therefore, our study is the first study to report an association between the history of macroscopic hematuria and a higher prevalence of both hypertension and elevated BP during long-term follow-up with higher recordings of office and ambulatory BP.

### Hematuria

Macroscopic hematuria tends to appear in children, similarly to adults with ADPKD, as a new marker of more severe disease. The reason for this association could be attributed to the increasing severity of cystic kidney involvement (higher number of renal cysts in our study and larger kidney size in adults). On the contrary, the increased prevalence of hypertension in children after macroscopic hematuria, cannot be attributed to decreasing kidney function, as the eGFR was similar in both groups with or without hematuria. One pediatric study showed that children with a history of macroscopic hematuria have larger kidneys [18]. In another single-center study, children with both VEO and non-VEO-ADPKD and a history of macroscopic hematuria had larger kidneys as an expression of more severe cystic involvement [18]. In our study, we found a difference in absolute (expressed in mm), but not in relative (expressed in SDS) kidney size between children with or without a history of macroscopic hematuria. This could be due to small numbers of children having hematuria. However, it has been demonstrated in several pediatric studies that not only kidney size and total kidney volume but also cyst number and cyst volume — findings suggestive of structural kidney deterioration — are closely related to BP and hypertension [22, 23]. Although there was no difference in relative kidney size due to small sample size, a clear increase in the number of kidney cysts was observed, suggesting that macroscopic hematuria is a marker of structural deterioration and increased severity of the kidney disease even in children.

### Kidney function, proteinuria

We could not find any differences in eGFR or proteinuria between children with or without a history of macroscopic hematuria. These findings corroborate the usually negative associations between proteinuria, hypertension, and cystic involvement in children with ADPKD [19, 24].

The limitations of the study are mainly the retrospective design of our study, and the inability to adjust the results for confounding factors such as age due to an extremely low number of hypertensive subjects. However, children with VEO-ADPKD who have short disease duration present severe disease manifestations showing that age and disease duration are unlikely to be related [25]. Further limitations are the lack of information about the age and etiology of the episodes of macroscopic hematuria in the majority of children, lack of total kidney volume measurements, the lack of information on the cystic involvement and age at developing hypertension, along with BP levels at the time of the macroscopic hematuria episodes. However, we used the same definition of hypertension as was used in the study by Martinez et al., namely, the use of antihypertensive drugs, to be able to compare the results of those studies [4]. In addition, we analyzed elevated blood pressure defined as systolic or diastolic blood pressure ≥ 75th percentile for age and height, which showed similar results. Another limitation is the low number of children who suffered from macroscopic hematuria which could be the reason of the lacking association between kidney size or nephromegaly and hematuria.

The strengths of this study are the considerable number of pediatric patients with ADPKD, investigated uniform investigation protocols, and the long-term follow-up after episodes of macroscopic hematuria.

## Conclusions

This large long-term retrospective cohort study has shown for the first time that macroscopic hematuria in children with ADPKD is associated with an increased risk of arterial hypertension or elevated blood pressure later in the course of the disease. We can conclude that in pediatric patients with ADPKD, similar to adult patients, a history of macroscopic hematuria is associated with a higher risk of developing hypertension or elevated blood pressure during follow-up, and therefore, they require closer long-term blood pressure monitoring.

## Data Availability

No datasets were generated or analysed during the current study.

## Abbreviations

ABPM: Ambulatory blood pressure monitoring
ADPKD: Autosomal dominant polycystic kidney disease
BP: Blood pressure
CKD: Chronic kidney disease
eGFR: Estimated glomerular filtration rate
KDIGO: Kidney disease improving global outcomes
MaHu: Macroscopic hematuria
VEO: Very early onset

## References

[1] Willey CJ, Blais JD, Hall AK, Krasa HB, Makin AJ, Czerwiec FS (2017) Prevalence of autosomal dominant polycystic kidney disease in the European Union. Nephrol Dial Transplant 32:1356–1363. 10.1093/ndt/gfw240 27325254 10.1093/ndt/gfw240 PMC5837385

[2] Kidney Disease: Improving Global Outcomes (KDIGO) ADPKD Work Group (2025) KDIGO 2025 clinical practice guideline for the evaluation, management, and treatment of autosomal dominant polycystic kidney disease (ADPKD). Kidney Int 107:S1–S239. 10.1016/j.kint.2024.07.009 39848759 10.1016/j.kint.2024.07.009

[3] Gordon CE, Garimella PS, Perrone RD, Miskulin DC (2025) Autosomal dominant polycystic kidney disease: Core curriculum 2025. Am J Kidney Dis 86:525–542. 10.1053/j.ajkd.2025.05.010 40844441 10.1053/j.ajkd.2025.05.010 PMC13563424

[4] Martínez V, Furlano M, Sans L, Pulido L, García R, Pérez-Gómez MV, Sánchez-Rodríguez J, Blasco M, Castro-Alonso C, Fernández-Fresnedo G, Robles NR, Valenzuela MP, Naranjo J, Martín N, Pilco M, Agraz-Pamplona A, González-Rodríguez JD, Panizo N, Fraga G, Fernández L, López MT, Dall’Anese C, Ortiz A, Torra R (2022) Autosomal dominant polycystic kidney disease in young adults. Clin Kidney J 16:985–995. 10.1093/ckj/sfac251 37260991 10.1093/ckj/sfac251 PMC10229292

[5] Boyer O, Gagnadoux MF, Guest G, Biebuyck N, Charbit M, Salomon R, Niaudet P (2007) Prognosis of autosomal dominant polycystic kidney disease diagnosed in utero or at birth. Pediatr Nephrol 22:380–388. 10.1007/s00467-006-0327-8 17124604 10.1007/s00467-006-0327-8

[6] Mekahli D, Guay-Woodford LM, Cadnapaphornchai MA, Goldstein SL, Dandurand A, Jiang H, Jadhav P, Debuque L (2024) Estimating risk of rapid disease progression in pediatric patients with autosomal dominant polycystic kidney disease: A randomized trial of tolvaptan. Pediatr Nephrol 39:1481–1490. 10.1007/s00467-023-06239-8 38091246 10.1007/s00467-023-06239-8 PMC10942936

[7] Ravine D, Walker RG, Gibson RN, Forrest SM, Richards RI, Friend K, Sheffield LJ, Kincaid-Smith P, Danks DM (1992) Phenotype and genotype heterogeneity in autosomal dominant polycystic kidney disease. Lancet 340:1330–1333. 10.1016/0140-6736(92)92503-8 1360045 10.1016/0140-6736(92)92503-8

[8] Flynn JT, Kaelber DC, Baker-Smith CM, Blowey D, Carroll AE, Daniels SR, de Ferranti SD, Dionne JM, Falkner B, Flinn SK, Gidding SS, Goodwin C, Leu MG, Powers ME, Rea C, Samuels J, Simasek M, Thaker VV, Urbina EM (2017) Clinical practice guideline for screening and management of high blood pressure in children and adolescents. Pediatrics 140:e20171904. 10.1542/peds.2017-1904 28827377 10.1542/peds.2017-1904

[9] Lurbe E, Agabiti-Rosei E, Cruickshank JK, Dominiczak A, Erdine S, Hirth A, Invitti C, Litwin M, Mancia G, Pall D, Rascher W, Redon J, Schaefer F, Seeman T, Sinha M, Stabouli S, Webb NJ, Wühl E, Zanchetti A (2016) 2016 European Society of Hypertension guidelines for the management of high blood pressure in children and adolescents. J Hypertens 34:1887–1920. 10.1097/HJH.0000000000001039 27467768 10.1097/HJH.0000000000001039

[10] Flynn JT, Urbina EM, Brady TM, Baker-Smith C, Daniels SR, Hayman LL, Mitsnefes M, Tran A, Zachariah JP (2022) Ambulatory blood pressure monitoring in children and adolescents: 2022 update: a scientific statement from the American Heart Association. Hypertension 79:e114–e124. 10.1161/HYP.0000000000000215 35603599 10.1161/HYP.0000000000000215 PMC12168719

[11] Schwartz GJ, Brion LP, Spitzer A (1987) The use of plasma creatinine concentration for estimating glomerular filtration rate in infants, children, and adolescents. Pediatr Clin North Am 34:571–590. 10.1016/s0031-3955(16)36251-4 3588043 10.1016/s0031-3955(16)36251-4

[12] Schwartz GJ, Muñoz A, Schneider MF, Mak RH, Kaskel F, Warady BA, Furth SL (2009) New equations to estimate GFR in children with CKD. J Am Soc Nephrol 20:629–637. 10.1681/ASN.2008030287 19158356 10.1681/ASN.2008030287 PMC2653687

[13] Kidney Disease: Improving Global Outcomes (KDIGO) CKD Work Group (2024) KDIGO 2024 clinical practice guideline for the evaluation and management of chronic kidney disease. Kidney Int 105(4):S117–S314. 10.1016/j.kint.2023.10.018 38490803 10.1016/j.kint.2023.10.018

[14] Obrycki Ł, Sarnecki J, Lichosik M, Sopińska M, Placzyńska M, Stańczyk M, Mirecka J, Wasilewska A, Michalski M, Lewandowska W, Dereziński T, Pac M, Szwarc N, Annusewicz K, Rekuta V, Ažukaitis K, Čekuolis A, Wierzbicka-Rucińska A, Jankauskiene A, Kalicki B, Jobs K, Tkaczyk M, Feber J, Litwin M (2022) Kidney length normative values in children aged 0–19 years - a multicenter study. Pediatr Nephrol 37:1075–1085. 10.1007/s00467-021-05303-5 34657197 10.1007/s00467-021-05303-5 PMC9023417

[15] Cadnapaphornchai MA, McFann K, Strain JD, Masoumi A, Schrier RW (2009) Prospective change in renal volume and function in children with ADPKD. Clin J Am Soc Nephrol 4:820–829. 10.2215/CJN.02810608 19346430 10.2215/CJN.02810608 PMC2666428

[16] Gabow PA, Duley I, Johnson AM (1992) Clinical profiles of gross hematuria in autosomal dominant polycystic kidney disease. Am J Kidney Dis 20:140–143. 10.1016/s0272-6386(12)80541-5 1496966 10.1016/s0272-6386(12)80541-5

[17] Helal I, Reed B, McFann K, Yan X-D, Fick-Brosnahan GM, Cadnapaphornchai M, Schrier RW (2011) Glomerular hyperfiltration and renal progression in children with autosomal dominant polycystic kidney disease. Clin J Am Soc Nephrol 6:2439–2443. 10.2215/CJN.01010211 21903987 10.2215/CJN.01010211 PMC3186452

[18] Shamshirsaz AA, Shamshirsaz A, Bekheirnia MR, Bekheirnia RM, Kamgar M, Johnson AM, McFann K, Cadnapaphornchai M, Nobakhthaghighi N, Haghighi NN, Schrier RW (2005) Autosomal-dominant polycystic kidney disease in infancy and childhood: progression and outcome. Kidney Int 68:2218–2224. 10.1111/j.1523-1755.2005.00678.x 16221221 10.1111/j.1523-1755.2005.00678.x

[19] Marlais M, Cuthell O, Langan D, Dudley J, Sinha MD, Winyard PJ (2016) Hypertension in autosomal dominant polycystic kidney disease: a meta-analysis. Arch Dis Child 101:1142–1147. 10.1136/archdischild-2015-310221 27288429 10.1136/archdischild-2015-310221

[20] Turczyn A, Krzemień G, Nguyen D, Smyk K (2025) Total kidney volume, hypertension, and deterioration of kidney function in children with early-stage ADPKD. J Clin Med 14:4498. 10.3390/jcm14134498 40648870 10.3390/jcm14134498 PMC12249612

[21] Benz EG, Hartung EA (2021) Predictors of progression in autosomal dominant and autosomal recessive polycystic kidney disease. Pediatr Nephrol 36:2639–2658. 10.1007/s00467-020-04869-w 33474686 10.1007/s00467-020-04869-w PMC8292447

[22] Seeman T, Dusek J, Vondrichová H, Kyncl M, John U, Misselwitz J, Janda J (2003) Ambulatory blood pressure correlates with renal volume and number of renal cysts in children with autosomal dominant polycystic kidney disease. Blood Press Monit 8:107–10. 10.1097/01.mbp.0000085762.28312.4a 12900587 10.1097/01.mbp.0000085762.28312.4a

[23] Cadnapaphornchai MA, Masoumi A, Strain JD, McFann K, Schrier RW (2011) Magnetic resonance imaging of kidney and cyst volume in children with ADPKD. Clin J Am Soc Nephrol 6:369–376. 10.2215/CJN.03780410 21115621 10.2215/CJN.03780410 PMC3052228

[24] Seeman T, Pohl M, John U (2018) Proteinuria in children with autosomal dominant polycystic kidney disease. Minerva Pediatr 70:413–417. 10.23736/S0026-4946.16.04404-2 30302987 10.23736/S0026-4946.16.04404-2

[25] Nowak KL, Cadnapaphornchai MA, Chonchol MB, Schrier RW, Gitomer B (2016) Long-term outcomes in patients with very early onset autosomal dominant polycystic kidney disease. Am J Nephrol 44:171–178. 10.1159/000448695 27548646 10.1159/000448695 PMC5098215

